# The effect of Aqua Stretching exercises and Pilates on pain, function and spine posture in patients with ankylosing spondylitis: a randomized controlled trial

**DOI:** 10.1186/s13102-022-00577-0

**Published:** 2022-10-21

**Authors:** Farzaneh Gandomi, Parviz Soufivand, Mozhgan Ezati, Mehran Salimi, Shirin Assar, Mehran Pournazari, Homayoun Abbasi

**Affiliations:** 1 Sports Injuries and Corrective Exercises Department, Sport Sciences Faculty, Razi University, Kermanshah, Iran; 2 Rheumatology Department, Clinical Research Development Center, Imam Reza Hospital, Kermanshah University of Medical Sciences, Kermanshah, Iran; 3 Sports Injuries and Corrective Exercises Department, Sport Sciences Faculty, Tehran University, Tehran, Iran; 4 Sports Management Department, Sport Sciences Faculty, Razi University, Kermanshah, Iran

**Keywords:** Ankylosing spondylitis, Aqua exercises, Stretch, Pilates, Pain, Function, Quality of life, Spine

## Abstract

**Background:**

Aqua Pilates and Aqua Stretch exercises are different and new methods for the rehabilitation of musculoskeletal disorders. This study aimed to compare the effectiveness of Aqua Stretch and Aqua Pilates interventions in the treatment of pain, function, and posture of the spine in ankylosing spondylitis (AS) patients.

**Methods:**

Forty patients participated in this study who were randomly allocated into Aqua Stretch, aqua Pilates, and control. The experimental groups received four 60-min training sessions each week for six weeks. However, the control group had only its routine drug treatment (NSAIDs & Anti TNF). Pain with Visual Analog Scale (VAS), function with Bath Ankylosing Spondylitis Functional Index (BASFI) and 40-m walking test (MWT), quality of life with ankylosing spondylitis quality of life (ASQoL), and posture of the spine with the Spinal Mouse were evaluated. Evaluations were performed before and after the interventions. Repeated measure ANOVA was employed to determine the main and interaction effects.

**Results:**

Aqua Stretch and Aqua Pilates had a significant effect on pain (Aqua-Pilates: *P* = 0.0001; Aqua-Stretch: *P* = 0.0001), BASFI (Aqua-Pilates: *P* = 0.01; Aqua-Stretch: *P* = 0.02), 40-MWT (Aqua-Pilates: *P* = 0.006; Aqua-Stretch: *P* = 0.0001) and ASQoL (Aqua-Pilates: *P* = 0.01; Aqua-Stretch: *P* = 0.001), spinal range of motion (ROM) (Aqua-Pilates: *P* = 0.0001; Aqua-Stretch: *P* = 0.0001) at a similar ratio. However, the control group did not present any improvement in these factors (*P* > 0.05). Moreover, the minimal clinically important difference (MCID) revealed that the Aqua Stretch group performed better than the Aqua Pilates group in terms of VAS, ASQOL, and 40-MWT factors.

**Conclusions:**

Aqua Stretch and Aqua Pilates had statistically the same effect on improving pain, function, quality of life, and spinal ROM, while MCID results revealed that the Aqua Stretch group performed better than the Aqua Pilates in terms of VAS-ASQOL-40-MWT.

*Trial registration* It is notable that local ethics committee approval was obtained (IR.KUMS.REC.1399.1137), and the study was registered in Iranian Registry of Clinical Trials (IRCT; IRCT20190426043377N3; registered on 22/05/2021, https://fa.irct.ir/user/trial/56058/view) and patient recruitments were started on 06/07/2021.

## Introduction

Ankylosing spondylitis (AS) is a chronic, progressive, inflammatory disease of unknown etiology, mainly characterized by pain, fatigue, joint destruction, deformity, disability, and joint dysfunction [1]. Current treatment of AS includes a combination of pharmacological and non-pharmacological treatments [2]. The prevalence of AS is generally believed to be between 0.1% and 1.4% globally [2]. A recent study reported that the mean AS prevalence per 10,000 populations was 23.8 in Europe, 16.7 in Asia, 31.9 in North America, 10.2 in Latin America, and 7.4 in Africa [3]. Even though the causes of AS have not yet been identified, evidence suggests the potential role of some factors in the prevalence of AS, such as heredity and environmental factors, including intestinal microorganisms and microbiomes [1].

While medications such as inhibitors of tumor necrosis factor-alpha (TNF-a) are suggested to improve the quality of life of patients with AS, non-pharmacological therapies, including exercise [4], manual therapy, physiotherapy, and education are recommended complementary therapies [5]. Exercise proposed for patients with AS can increase their muscles strength and flexibility and improve lung function [6, 7]. Some researchers have suggested the aqua-based exercises for this patients [8]. Generally, the natural resistance of the water strengthens the muscles. In addition, the drag forces may decrease mechanical loads on spinal joints [9]. Water buoyancy forces, also, lessens stress on the joints, bones, and muscles, facilitates movement, and may decrease the pain by affecting thermal receptors and mechanoreceptors [9]. These results show the potential benefits of aqua-based exercises for the patients with AS [10]. Unfortunately, few studies are conducted in this field. Given the positive effect of stretch exercises on the spine muscles of men and women with AS disease to increase lung volume [6], and the risks of stretch exercises on land, transfer of these exercises to an aquatic environment can improve their effects on the spine posture and pain of patients with AS.

Pilates exercises are other types of intervention used to improve the conditions of patients with AS [11]. This type of exercise has recently become popular in rehabilitation programs towing to its perceived advantages [12–14]. Nevertheless, given the conditions of patients with AS and the difficulty of training on land, the effects of Pilates exercises can be improved by laying a proper foundation for doing them in an aquatic environment that no study has been done in this field so far.

Regarding the benefits of making physical exercises easier for patients with AS in aquatic environments, and given the advantages of the foregoing interventions (Pilates and stretching) and their effectiveness in patients' rehabilitation, the present study’s objective was the evaluation of the effectiveness of aqua-based Pilates versus stretch exercise programs on pain, physical function, spinal mobility, and quality of life of patients with AS.

## Material and methods

### Participants

This three parallel groups, randomized, controlled and assessor blind study was conducted in Sport Injuries and Corrective Exercises Department of Razi University and Rheumatology Department, Clinical Research Development Center, Imam Reza Hospital. Patients (all the patients were men) with 1988 modified New York criteria [15] for AS were enrolled in the study and assigned to three groups. In addition to their demographic characteristics including age, weight, height, and body mass index (BMI), they were also to recount the presence or absence of the main symptoms, time of diagnosis, and drug usage status. Exclusion criteria were the presence of prosthesis, hypertension, cardiovascular disease, chronic obstructive pulmonary disease, and exercising regularly during the previous three months [16]. Patients whose treatment regimens had changed during the last two months prior to the study were not included. Additionally, patients who were not vaccinated with two doses of Covid-19 vaccination were excluded. The included patients were asked not to use supplementary drugs or change the usual dosages throughout the study period. For a more accurate pain assessment, they were asked not to take any analgesics in the morning of the assessment day. The participants were fully informed about the nature and purpose of the study, and written informed consent was obtained from all subjects. It is notable that local ethics committee approval was obtained (IR.KUMS.REC.1399.1137), and the study was registered in Iranian Registry of Clinical Trials (IRCT20190426043377N3, registered on 22/05/2021, https://fa.irct.ir/user/trial/56058/view). patient recruitments were also started on 06/07/2021.

**Fig. 1:**
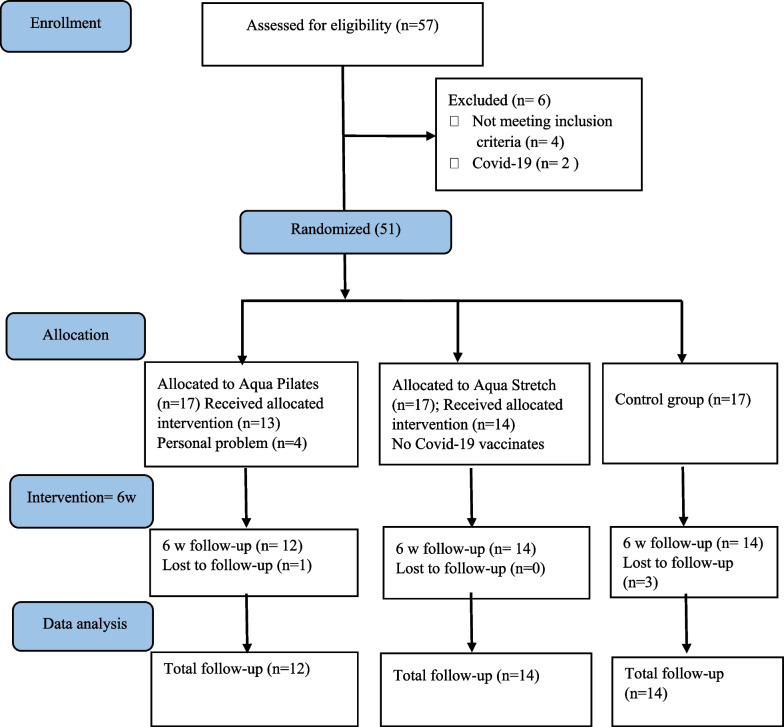
CONSORT flow diagram

G ∗ power statistical software (Heinrich Heine University, Düsseldorf, Germany) was used to determine the sample size for a repeated-measures analysis of variance at 90.0% power, an effect size of 0.31 [17], and a 5.0% level of significance. As a result, the minimum number of patients required in the Aqua Stretch, aqua Pilates, and control group was estimated to be at least 13 patients. Dropping out rate was considered 20%. The patients were randomly assigned to three groups: aqua-based stretch (n = 17), aqua-based Pilates (n = 17), and a control group (n = 17) (Fig. 1). A software tool (Random Number Generator) was dedicated to simple randomly generating sequences, and the corresponding numbers were provided in sealed, opaque envelopes opened upon each patient’s agreement to participate in the research. Concealment of allocation was done by assigning the allocation task to a research assistant who was not involved in the recruitment process. Participants were randomly assigned to the Aqua Pilates and Aqua Stretch groups or no treatment (control) group in a 1:1:1 ratio. Eligible participants were informed of their allocation result by a project manager following the assessment of their baseline measurements. In this study, blinding of the outcome assessors and the statistician was maintained even though it was impossible to blind the participants and exercise coaches.

### Interventions

All patients of the intervention groups were trained under the supervision of a Corrective Exercises and Sports Injuries specialist. He was a certified Pilates and aquatic therapy practitioner with 5 years of experience.

#### Aqua-based Stretch program

In total, 24 Aqua Stretch exercise sessions were held (four sessions per week), lasting for six weeks. Exercises were performed in a swimming pool at 32–33°c, and each session lasted 60 min. The program started with 10–15 min of poolside exercises including warming-up (forward, backward, and sideward walking and jogging). Self-wall side passive stretching exercises, executed by noodles, were done for 30-min during the first two weeks, followed by self-wall side active stretching exercises were performed in 35-min sessions for the second two weeks, and in 45-min sessions for the third two weeks. Each stretching exercise lasted 15 s, 30 s, and 45 s for the first, second, and third two weeks, respectively. Stretching exercises were performed in two series; the first series of stretches included stretching exercises performed on each joint [18, 19]. The second series of stretches was performed based on the pattern of muscle chains [20, 21]. The program ended with cooling-down including slow walking and passive stretching for 5–15 min.

#### Aqua-based Pilates program

In this study, a total of 24 sessions of aqua-based Pilates exercises was performed (four sessions per week), lasting for six weeks. The exercises were performed in a swimming pool at 32–33 °C, and each session lasted 60 min. The program started with 10 min poolside exercises including warming-up (five min of walking and five min of general active range of motion (ROM) and stretching). Poolside exercises were followed by 45 min of main exercises such as: breathing exercises, standing criss cross, T balance with noodle, standing spine stretch (3 set, 20 s hold), spine twists, standing single kicks (3 set, 10 repetition) for the first two weeks. Breathing, standing spine stretch and single side leg lift, plank on a noodle, side kick, hip work, single leg stretch (by cycling on a noodle), T balance exercises (3 set, 10 repetition) for the second two weeks. Finally, breathing and side kick exercises, side plank with one hand on a noodle, spine twist (3 set, 10 repetition), cat stretch and T balance in water perturbation (3 set, 20 s hold). The program ended with cooling-down, including slow walking and passive stretching for five min [22].

Meanwhile, the control group patients continued their usual activities and drug treatment (NSAIDs & Anti TNF).

### Outcome measures

The patients were assessed in terms of pain, spinal mobility, physical function, and quality of life. Evaluations were performed before (week 0) and after (week 6) treatment. The research assessor was blinded since they assessed the patients’ clinical assessment parameters prior to their assignment to groups. In addition, spinal mobility of the subjects was assessed by Spinal Moues and physical function was evaluated by The Bath Ankylosing Spondylitis Functional Index (BASFI) and 40-m walking Test (MWT). Pain was also assessed by 0–10 cm visual analog scale (VAS). Finally, quality of life was evaluated by Ankylosing spondylitis quality of life (ASQoL) questionnaire.

#### Pain

In this study, the VAS was used to measure the intensity of perceived pain. The mentioned tool is a ruler in the form of a horizontal strip with the length of 10 cm, on which one end is zero (no pain) and the other end is 10 (excruciating pain). The ruler also has two qualitative and quantitative sides. In the present study, the patients were asked to mark the qualitative side of the ruler based on their perceived pain intensity. Following that, the marked point was recorded by the researcher as the pain rate of patients [23]. The ICC (95% CI 0.96 to 0.98) of this scale was reported to be 0.97 [24].

#### Functional capacity

The BASFI is composed by ten questions elaborated to determine the degree of functional limitation in patients with AS. The participants were asked to indicate their level of ability within the last week on a 10-cm horizontal line (from easy to impossible). The BASFI sum score was calculated by adding all scores from questions 1–10 and then dividing them by 10 (ICC was reported 0.94) [25, 26]. In addition, the 40-MWT was used to assess functional capacity [27]. For this purpose, the duration of walking a distance of 40 m was recorded with a stopwatch in seconds. A shorter duration indicated greater physical fitness (ICC = 0.99) [28].

#### Health quality

The ASQOL questionnaire is an instrument specific for patients with AS [29]. The tool consists of 18 items requesting a yes or no response to questions. All item scores are summed to give a total score or index which may range from 0 (good QoL) to 18 (poor QoL). The correlation coefficient for test–retest reliability of the ASQOL questionnaire was 0.85 (95% CI 0.80–0.89) [30].

#### Spine posture evaluation

A spinal mouse device (model: 3.32, made in Switzerland) was used to measure the spinal curves angles at the sagittal plane (ICC: 0.63–0.97) [31]. The spinal mouse is a computer-assisted device, with which spinal shape and mobility is assessed with surface-based measurements. In this test, the participants stood in front of the assessor with bare feet, and the C7 and S2 landmarks were marked. In the next stage, the angle of the thoracic kyphosis and lumbar lordosis in the upright position were measured by placing the mouse device on the C7 vertebra and pulling down the vertebral spinous. The flexion and extension range of motion (ROM) was also measured in the upright position. Moreover, thoracic kyphosis, lumbar lordosis, and sacral inclination were measured in the next stage. Thoracic kyphosis refers to forward curvature of the thoracic spine in the sagittal plane, from the first thoracic vertebra (T1) to the 12th thoracic vertebra (T12) and the sum of the 11 angles from T1/2 to T11/12. On the other hand, the lumbar lordosis angle is the sum of the six angles from T12/lumbar 1 to lumbar 5/sacral 1 (S1) in the curvature throughout the lumbar spine (T12-S1). Finally, sacral slope (SS) is defined as the angle between the horizontal and the sacral plate, drawn along the segmented endplate of the sacrum. In this study, the positive values obtained from the measurements were attributed to kyphosis, whereas the negative values indicated lordosis [32].

#### Statistical analysis

Statistical analysis was performed in SPSS version 22.0.0 (IBM Corporation, Armonk, NY, USA). In addition, means and standard deviations were calculated for all dependent variables. The Shapiro-Wilks test was applied to test the normality of quantitative data, and One-way ANOVA test was used to assess differences between the groups at baseline and compare the mean differences of variables among groups. Moreover, repeated measure ANOVA was employed to determine the main and interaction effects of Time (pretest vs. posttest) and Group (Aqua Stretch, Aqua Pilates, and control) on the outcome measures. Bonferoni test was used for post-hoc analysis when necessary. Notably, paired sample t-test was used to investigate the within-group comparisons in case of significant interaction effects. Furthermore, one-way ANOVA was used to evaluate the between-group comparisons. It is notable that *P* ≤ 0.05 was considered statistically significant.

Based on the 95% confidence interval in the current study, the minimal clinically important difference (MCID) was assessed using the distribution-based approach and the reliable change index (RCI), while the minimal detectable change (MDC) was evaluated using the standard error of measurement (SEM) and the following equations [33]:

$${\text{SEM}} = {\text{SD}}_{{{\text{pre}}}} \surd {1} - {\text{r}}_{{{\text{test}}}}$$

$${\text{MDC}}_{{\% {95}}} = {1}.{96} \times \surd {2}\;{\text{SEM}}$$

$${\text{MCID}}_{{{\text{RCI}}}} = {1}.{96} \times {\text{SD}}_{{{\text{pre}}}} \left\{ {\surd [{2} \times ({1} - {\text{r}}_{{{\text{test}}}} )]} \right\}$$

## Results

### Baseline characteristics

As shown in Fig. 1, 51 out of 57 patients screened were enrolled and randomly assigned to one of the three groups. However, four participants in Aqua Pilates group, three participants in Aqua Stretch group and three participants in control group were unwilling to participate in interventions, and one of the subjects in the Aqua Pilates group was unable to participate in follow-up assessments. Therefore, a total of 40 participants (n_aqua Pilates_ = 14, n_aqua stretch_ = 12, n_control_ = 14) were included in the final data analysis. The demographic characteristics of the patients and pretreatment evaluation parameters are presented in the Table 1. The three research groups were similar in terms of the demographic and baseline characteristics.

**Table 1:** Comparison of demographic characteristics and ankylosing spondylitis symptoms between Aqua Stretch, Aqua Pilates exercise and no treatment groups at baseline

Characteristics and parameters	Aqua Pilates (n = 12)	Aqua Stretch (n = 14)	Control (*n* = 14)	*P*-value
	M (SD)	M (SD)	M (SD)	
Age (yr.)	42.58 (14.18)	39.21 (10.25)	38.07 (8.69)	0.57
Height (cm)	173.16 (8.53)	171.85 (6.45)	172.78 (8.01)	0.90
Weight (kg)	84.66 (8.53)	75.61 (10.11)	79.17 (12.32)	0.14
BMI (kg/m^2^)	28.20 (3.73)	25.53 (2.76)	26.48 (3.42)	0.13
BASFI (0–10)	3.41 (2.72)	3.24 (2.24)	4.12 (2.39)	0.60
ASQoL (0–18)	7.50 (5.66)	10.35 (5.40)	6.30 (4.92)	0.12
VAS (0–10)	4.58 (2.42))	4.78 (2.45)	5.78 (2.32)	0.39
40-MWT (s)	39.57 (9.89)	40.48 (6.14)	39.14 (9.84)	0.92
Disease duration (yr.)	11.25 (7.60)	10.42 (10.76)	7.42 (4.89)	0.45
Drug using duration (yr.)	7.27 (5.21)	7.42 (9.75)	6.30 (4.93)	0.91

BMI, body mass index; BASFI, the bath ankylosing spondylitis functional index; ASQoL, ankylosing spondylitis quality of life; VAS, visual analog scale; M(SD), mean (standard deviation); 40-MWT, 40 m walking test

### Outcome variables

There were statistically significant group-by-time effects for VAS, BASFI and 40-MWT (Table 2). In the aqua-based stretch group, there were statistically significant differences in VAS, BASFI and 40-MWT regarding baseline and post-intervention (*P* = 0.001, *P* = 0.021 and *P* = 0.0001, respectively). In the aqua-based Pilates group, there were statistically significant differences in VAS, BASFI and 40-MWT regarding baseline and post-intervention (*P* = 0.0001, *P* = 0.014 and *P* = 0.006). Notably, this improvement in VAS and 40-MWT was higher in aqua-based stretch group, compared to the aqua-based Pilates. In the control group, however, there were no statistically significant differences in VAS, BASFI and 40-MWT in terms of baseline and post-intervention (*P* = 0.38, *P* = 0.58 and *P* = 0.26, respectively) (Table 2). Comparison of mean differences for pain also showed a significant difference between groups (F(2,37) = 16.04, *P* = 0.0001). Based on the Bonferroni post-hoc test results, there were significant differences between Aqua Stretch and control groups (*P* = 0.0001), and between aqua-based Pilates and control groups (*P* = 0.0001). However, there were no significant differences between Aqua Stretch and Aqua Pilates groups (*P* = 1.00). Additionally, there were significant differences among the groups in terms of mean differences of 40-MWT (F(2,37) = 6.06, *P* = 0.005). Bonferroni post-hoc test results showed that there were significant differences between Aqua Stretch and control groups (*P* = 0.007), and between Aqua Pilates and control group (*P* = 0.04). Meanwhile, no significant differences were found between Aqua Stretch and Aqua Pilates group (*P* = 1.00).

**Table 2:** Comparison of outcome measures (baseline to six-week follow-up)

Outcome measures	Groups	Baseline M ± SD	Six weeks M ± SD	*P*-value^1^	*P*-value^2^ (η^2^) Time/Time × Group
VAS (0–10)	Aqua Pilates (n = 12)	4.58 ± 2.42	1.75 ± 1.28	0.0001**	
	Aqua Stretch (n = 14)	4.78 ± 2.45	1.78 ± 1.57	0.0001**	0.0001** (0.49)/0.005** (0.24)
	Control (n = 14)	5.78 ± 2.32	6.14 ± 2.31	0.38	
BASFI (0–10)	Aqua Pilates (n = 12)	3.41 ± 2.72	1.80 ± 1.74	0.014*	
	Aqua Stretch (n = 14)	3.24 ± 2.24	1.77 ± 1.59	0.021*	0.001**(0.25)/0.020*(0.19)
	Control (n = 14)	4.12 ± 2.39	4.27 ± 2.43	0.58	
40-MWT (s)	Aqua Pilates (n = 12)	39.57 ± 9.89	34.93 ± 6.06	0.006**	
	Aqua Stretch (n = 14)	40.48 ± 6.14	35.01 ± 4.43	0.0001**	0.0001** (0.54)/0.0001** (0.46)
	Control (n = 14)	39.14 ± 9.84	38.40 ± 9.17	0.26	
ASQoL (0–18)	Aqua Pilates (n = 12)	7.50 ± 5.66	3.66 ± 4.22	0.015**	
	Aqua Stretch (n = 14)	10.35 ± 5.40	5.85 ± 4.76	0.001**	0.0001** (0.24)/0.36 (0.05)
	Control (n = 14)	12.00 ± 6.71	10.07 ± 6.71	0.23	
*Spinal posture (standing)*					
Kyphosis (°)	Aqua Pilates (n = 12)	45.58 ± 10.56	47.71 ± 11.79	0.7	
	Aqua Stretch (n = 14)	45.35 ± 10.62	47.71 ± 11.76	0.35	0.81(0.001)/0.63(0.025)
	Control (n = 14)	48.14 ± 12.40	45.85 ± 10.56	0.62	
Lordosis (°)	Aqua Pilates (n = 12)	− 16.58 ± 11.21	− 16.75 ± 12.84	0.93	
	Aqua Stretch (n = 14)	− 13.28 ± 14.33	− 15.21 ± 14.80	0.4	0.54(0.01)/0.93(0.0004)
	Control (n = 14)	− 14.71 ± 12.66	− 16.42 ± 13.26	0.74	
Incline (°)	Aqua Pilates (n = 12)	6.62 ± 7.69	6.91 ± 6.02	1	
	Aqua Stretch (n = 14)	5.57 ± 6.06	4.71 ± 5.95	0.21	0.41 (0.018)/0.78 (0.013)
	Control (n = 14)	7.42 ± 7.83	5.42 ± 7.07	0.53	
*Spinal ROM (flexion/extension)*					
Kyphosis (°)	Aqua Pilates (n = 12)	17.08 ± 10.56	17.58 ± 13.66	0.86	
	Aqua Stretch (n = 14)	25.00 ± 12.65	21.00 ± 17.15	0.38	0.67(0.005)/0.17(0.09)
	Control (n = 14)	16.21 ± 12.96	22.57 ± 10.62	0.13	
Lordosis (°)	Aqua Pilates (n = 12)	33.50 ± 21.42	37.25 ± 24.59	0.057	
	Aqua Stretch (n = 14)	45.35 ± 10.62	47.71 ± 11.76	0.35	0.36(0.02)/0.90(0.005)
	Control (n = 14)	32.78 ± 20.35	34.00 ± 20.16	0.89	
Inclination (°)	Aqua Pilates (n = 12)	97.91 ± 24.53	114.75 ± 27.17	0.0001**	
	Aqua Stretch (n = 14)	108.85 ± 30.65	127.42 ± 31.77	0.0001**	0.07(0.08)/0.16 (0.09)
	Control (n = 14)	97.21 ± 34.01	92.42 ± 36.73	0.75	

M, mean; SD, standard deviation; VAS, visual analog scale; ROM, range of motion; BMI, body mass index; ASQoL, ankylosing spondylitis quality of life; MWT, meter walking test; BASFI, the bath ankylosing spondylitis functional index

*P*-value^1^: paired sample *t*-test; *P*-value^2^: 3 × 2 repeated measures mixed-model ANOVA; values expressed as mean ± standard deviation; figures in parentheses show effect sizes; *P < 0.05; **P < 0.01

There was no statistically significant time-by-group effect for ASQoL (*P* = 0.36), whereas there were statistically significant time and group effects for ASQoL (*P* = 0.0001, *P* = 0.025, respectively). Moreover, there were statistically significant differences in ASQoL between baseline and post-intervention for Aqua Stretch and Aqua Pilates (*P* = 0.001 and *P* = 0.015, respectively). However, there were no statistically significant differences in ASQoL between baseline and post-intervention for control group (*P* = 0.23). Additionally, there were significant differences among mean differences of ASQoL of groups (F(2,37) = 6.69, *P* = 0.003). Bonferroni post-hoc test results showed that there were significant differences between Aqua Stretch and control groups (*P* = 0.005). Furthermore, there were significant differences between Aqua Pilates and control groups (*P* = 0.024). Nevertheless, there were no significant differences between Aqua Stretch and Aqua Pilates groups (*P* = 1.00).

According to the results, there were no statistically significant time, time-by-group, and group effects for posture of spine variables and spine ROM (*P* > 0.05) (Table 2). However, there were statistically significant differences in spinal ROM between baseline and post-intervention for Aqua Stretch and Aqua Pilates groups (*P* = 0.0001 and *P* = 0.0001, respectively). However, there were no statistically significant differences for control group in this regard (*P* = 0.75).

**Fig. 2:**
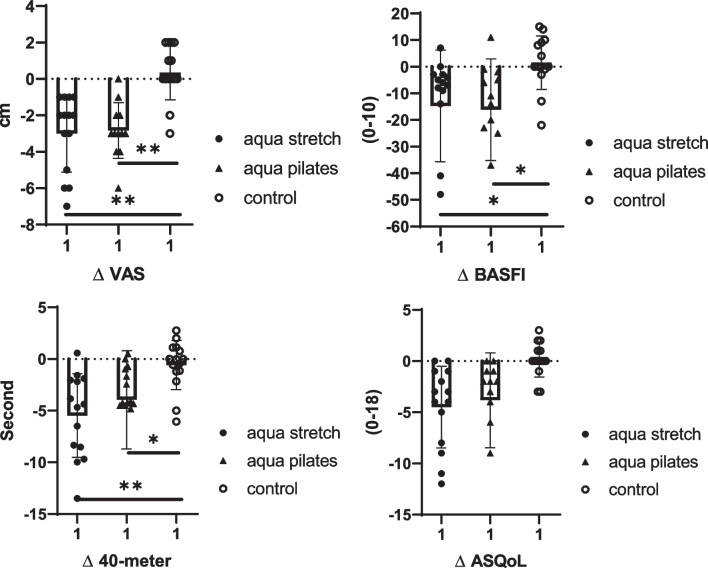
Pairwise comparison for pain, ASQoL, 40-MWT, and BASFI (Δ: posttest − pretest; *P < 0.05 and **P < 0.01)

Moreover, Fig. 2 depicts the pair comparisons of delta scores of study variables (posttest scores minus pretest scores) between the groups.

Moreover, we calculated the MCIDs for all variables to assess which one of exercise intervention is more effective clinically for patients of this study. There are two important conditions in the clinical examination of a study. First, an MCID is valid when its value is at least equal to or higher than that of the MDC. This condition holds in the current study. Second, the mean difference (∆ score) of the intervention group must be higher than the MCID so that it can be said with certainty that the intervention has been effective on the desired variable [33]. This condition also holds in the current study.

The investigation of the ∆ scores of the variables in the intervention groups and their comparison with the MCID showed that the Aqua Stretch exercises outperformed the Aqua Pilates exercises in terms of pain ((∆ scores <=> MCID): for Aqua Stretch: 3 > 1.50; for Aqua Pilates: 2.83 > 1.50), 40-MWT ((∆ scores <=> MCID): for Aqua Stretch: 5.47 > 4.97; for Aqua Pilates: 4.64 < 4.97), and ASQoL ((∆ scores <=> MCID): for Aqua Stretch: 4.51 > 2.64; for Aqua Pilates: 3.84 < 2.64) variables. However, Aqua Pilates exercises outperformed the Aqua Stretch exercises in terms of BASFI ((∆ scores <=> MCID): for Aqua Stretch: 1.47 < 1.52; for Aqua Pilates: 1.61 > 1.52) (Table 3).

**Table 3:** The MDC and MCID values of the study variables

Variable	SD_pre_ intervention groups	∆ Score (Aqua Pilates)	∆ Score (Aqua Stretch)	SEM	MDC_%95_	MCID (RCI)
VAS (0–10)	2.39	2.83	3.00	0.52	1.44	1.50
BASFI (0–10)	2.43	1.61	1.47	0.53	1.46	1.52
40-MWT (s)	7.93	4.64	5.47	1.74	1.74	4.97
ASQoL (0–18)	5.60	3.84	4.51	0.95	0.95	2.64
Inclination ROM	28.01	16.84	18.57	12.32	33.81	34.58

∆ score: (mean_pre_ − mean_post_)

SEM, standard error of measurement; MDC_%95_, minimal detectable change; MCID, minimal clinically important difference; RCI, reliable change index; SD, standard deviation; VAS, visual analog scale; BMI, body mass index; ASQoL, ankylosing spondylitis quality of life; ROM, range of motion

## Discussion

According to the results of this study, there were no significant differences between the Aqua Pilates and Aqua Stretch groups regarding the decrease of pain in patients with AS, which is consistent with the results of previous studies [1, 34, 35]. In fact, researchers assumed that two differently designed aqua exercise programs (stretching and Pilates exercises) would have different effect on patients’ pain and function, which was not approved. Remarkable water properties, such as warmth, weight reduction, buoyancy force, and hydrostatic pressure of water molecules, create a safe environment for performing exercises by patients [36]. These features also considerably affect patients’ pain regardless of the nature of exercises. The warmth and buoyancy of water may block pain receptors and improve proprioception by affecting thermal receptors and mechanoreceptors [34]. Furthermore, hydrostatic pressure in water reduces tissue swelling and pain by applying forces perpendicular to the joints of the spine [37]. In this respect, our findings are congruent with the results obtained by Bestaş et al. (2022), who mentioned the positive effects of aquatic exercises on the improvement of pain and function of patients with AS [35]. In a meta-analysis, Zhao et al. reported the significant effects of aquatic exercises on the pain reduction in patients with AS, claiming that more clinical trials should be performed in this area [38]. In addition, the results obtained by Dundar et al. were indicative of the positive effects of aquatic exercises on the reduction of pain and improvement of quality of life in patients with AS, claiming that aquatic exercises had more positive effects on pain reduction and life quality improvement of patients with AS, compared to exercising on land [37].

In the current research, the performance capacity of patients was assessed by the BASFI instrument and the 40-MWT. In the end, no significant differences were observed between Aqua Stretch and Pilates groups, which might be due to a similar impact on pain decrease and improvement of muscle performance in the participants of both groups. Regardless of the type of exercise given in the aquatic environment, the warmth of the water, comfortable and painless exercises, and hydrostatic pressure in water have been able to reduce time to walk a distance of 40-MWT and improve the functional capacity of patients by decreasing the pain, strengthening the muscles and increasing the range of motion of the joints, which confirms the results of studies performed in this area. In a meta-analysis, Zhao et al. [38] evaluated the effect of aqua-based physical therapy on the activity and function of patients with AS, reporting that aquatic exercises can significantly reduce pain and improve performance. In a prospective controlled study, Bestaş et al. [35] performed a comparative assessment of aqua-based and land-based exercises to determine their effects on the function of patients with AS. In the end, it was concluded that both types of exercise affected the pain and function capacity of patients. In another study, researchers compared swimming and traditional exercises in terms of function improvement in patients with AS, reporting that aerobic exercises (e.g., swimming) had double effects on the improvement of functional capacity of patients [8]. Nonetheless, in a systematic review and meta-analysis, Liang et al. [1] pointed out that “hydrotherapy could reduce pain in patients with AS but cannot enhance their functional capacity or spinal mobility”, which is inconsistent with our findings. This lack of consistency might be due to the use of different performance assessment tools.

According to the results of the present study, both groups significantly improved the inclination range of motion. In fact, the exercises performed in both groups improved spinal mobility by using stretching and breathing exercises and applying the mentioned water properties. Nonetheless, the Aqua Stretch group had a higher effect, compared to the Aqua Pilates group, which might be related to the nature of Aqua Stretch exercises regarding the improvement of articular function and muscle chains. Obviously, doing stretching exercises reduces muscle stiffness and joint capsules. Unfortunately, few studies have assessed the effectiveness of aquatic exercises on spinal improvement. Consistent with our findings, Dundar et al. mentioned the positive effects of aqua-based exercises on the improvement of spinal range of motion. However, the mentioned research evaluated the range of motion by an inclinometer, whereas it was assessed by a Spinal Mouse device in the current research [37].

We also assessed the effect of Aqua Stretching and Pilates on the improvement of the quality of life of patients with AS. Based on our findings, both interventions had significant effects on the improvement of patients’ quality of life, which was higher in the Aqua Stretch group. Aqua Stretching exercises may provide patients with a more desirable feeling by reducing the stiffness of the muscle–tendon–ligament complex and the joint capsule, resulting in greater spinal mobility. Significant improvement in quality of life was expected due to the reduction of pain and improvement of physical function of patients in both groups, which is congruent with the results of previous studies. Similarly, Dundar et al. reported that aqua-based exercises had better effects on pain and quality of life of patients with AS, compared to exercises on land [37]. In another research, Bestaş et al. mentioned the effectiveness of aqua-based exercises on the improvement of the quality of life of patients with AS [35].

By observing no significant difference between the intervention groups in the current study and clinical changes that are important in RCT studies, minimal detectable change (MDC) and minimal clinically important difference (MCID) findings can be used. Based on the results of MDC and MCID, it was observed that the Aqua Stretch exercises outperformed the Aqua Pilates exercises in terms of pain, 40-MWT, and ASQoL variables (Table 3). This may be because stretching exercises improve muscle stiffness in these patients [39], followed by flexibility, and reduce swelling and pain [6, 40].

There are several limitations to our study. Firstly, there was lack of a control group for land-based stretch and Pilates exercises. Randomized controlled trials comparing aqua-based stretch and Pilates exercises versus land-based stretch and Pilates interventions should be encouraged to determine the cumulative or additive benefits of these different environments on the physical and well-being of participants with AS. Secondly, we were unable to evaluate these issues in female patients due to the laws of Islamic countries. Thirdly, measurement of presence and/or absence of pain symptoms was not done at every session. Finally, the study was based on a relatively small number of participants who completed the aquatic exercise interventions. Future studies with larger sample sizes are needed to support or refute our findings. Another important limitation of this study is the short-term follow-up of 6 weeks (at the end of the intervention). This study does not maintain the effects of the intervention after the end of the study. Researchers are suggested to investigate this point in future research.

## Conclusion

According to the results of the present study, six weeks of aqua-based Pilates and stretch exercise programs (four sessions a week) significantly reduced pain and improved performance capacity, quality of life and spinal mobility of patients with AS and had great potential for improvement of patients’ conditions. Additionally, the results of MCID and MDC revealed that the Aqua Stretch group performed better than the Aqua Pilates group in terms of VAS-ASQOL-40-MWT walking factors. Therefore, it is recommended that aquatic exercises be used by sports experts, therapists and physical therapists as the first part of a therapeutic exercise program for patients with AS.

## Data Availability

The datasets generated and/or analysed during the current study are not publicly available but are available from the corresponding author on reasonable request.

## Abbreviations

AS: Ankylosing spondylitis
BMI: Body mass index
ROM: Range of motion
VAS: Visual analog scale
BASFI: The bath ankylosing spondylitis functional index
MWT: Meter walk test
ASQoL: Ankylosing spondylitis quality of life

## References

[1] Liang H, Xu L, Tian X, Wang S, Liu X, Dai Y (2020). The comparative efficacy of supervised-versus home-based exercise programs in patients with ankylosing spondylitis: a meta-analysis. Medicine.

[2] Akkoc N (2008). Are spondyloarthropathies as common as rheumatoid arthritis worldwide? A review. Curr Rheumatol Rep.

[3] Dean LE, Jones GT, MacDonald AG, Downham C, Sturrock RD, Macfarlane GJ (2014). Global prevalence of ankylosing spondylitis. Rheumatol.

[4] Falkenbach A (2003). Disability motivates patients with ankylosing spondylitis for more frequent physical exercise. Arch Phys Med Rehabil.

[5] Braun J (2018). Axial spondyloarthritis including ankylosing spondylitis. Rheumatology.

[6] Pécourneau V, Degboé Y, Barnetche T, Cantagrel A, Constantin A, Ruyssen-Witrand A (2018). Effectiveness of exercise programs in ankylosing spondylitis: a meta-analysis of randomized controlled trials. Arch Phys Med Rehabil.

[7] Basakci Calik B, Pekesen Kurtca M, Gur Kabul E, Telli Atalay O, Taskin H, Yigit M (2021). Investigation of the effectiveness of aerobic exercise training in individuals with ankylosing spondylitis: randomized controlled study. Mod Rheumatol.

[8] Karapolat H, Eyigor S, Zoghi M, Akkoc Y, Kirazli Y, Keser G (2009). Are swimming or aerobic exercise better than conventional exercise in ankylosing spondylitis patients? A randomized controlled study. Eur J Phys Rehabil Med.

[9] Bender T, Karagülle Z, Bálint GP, Gutenbrunner C, Bálint PV, Sukenik S (2005). Hydrotherapy, balneotherapy, and spa treatment in pain management. Rheumatol Int.

[10] Zão A, Cantista P (2017). The role of land and aquatic exercise in ankylosing spondylitis: a systematic review. Rheumatol Int.

[11] Rodríguez-López ES, Garnacho-Garnacho VE, Guodemar-Pérez J, García-Fernández P, Ruiz-Lopez M (2019). One year of pilates training for ankylosing spondylitis: a pilot study. J Altern Complement Med.

[12] La Touche R, Escalante K, Linares MT (2008). Treating non-specific chronic low back pain through the pilates method. J Bodyw Mov Ther.

[13] Donzelli S, Di Domenica F, Cova A, Galletti R, Giunta N (2006). Two different techniques in the rehabilitation treatment of low back pain: a randomized controlled trial. Eura Medicophys.

[14] Altan L, Korkmaz N, Dizdar M, Yurtkuran M (2012). Effect of pilates training on people with ankylosing spondylitis. Rheumatol Int.

[15] Sallam RA, Elbahnasawy AS (2020). Health related quality of life (HRQoL) in ankylosing spondylitis patients: relation to clinical features, disease activity and radiographic damage. Egypt Rheumatol.

[16] Durmuş D, Alaylı G, Uzun O, Tander B, Cantürk F, Bek Y (2009). Effects of two exercise interventions on pulmonary functions in the patients with ankylosing spondylitis. Joint Bone Spine.

[17] Lim JM, Cho O-H (2021). Effects of home-and-workplace combined exercise for patients with ankylosing spondylitis. Asian Nurs Res.

[18] Masiero S, Bonaldo L, Pigatto M, Nigro AL, Ramonda R, Punzi L (2011). Rehabilitation treatment in patients with ankylosing spondylitis stabilized with tumor necrosis factor inhibitor therapy. A randomized controlled trial. J Rheumatol.

[19] Kisner C, Colby LA, Borstad J. Therapeutic exercise: foundations and techniques: Fa Davis; 2017.

[20] Fernández-de-Las-Peñas C, Alonso-Blanco C, Alguacil-Diego IM, Miangolarra-Page JC (2006). One-year follow-up of two exercise interventions for the management of patients with ankylosing spondylitis: a randomized controlled trial. Am J Phys Med Rehabil.

[21] Fernández-de-Las-Peñas C, Alonso-Blanco C, Morales-Cabezas M, Miangolarra-Page JC (2005). Two exercise interventions for the management of patients with ankylosing spondylitis: a randomized controlled trial. Am J Phys Med Rehabil.

[22] Roşu MO, Ţopa I, Chirieac R, Ancuta C (2014). Effects of Pilates, McKenzie and Heckscher training on disease activity, spinal motility and pulmonary function in patients with ankylosing spondylitis: a randomized controlled trial. Rheumatol int.

[23] Sivas F, Başkan BM, Inal EE, Aktekin LA, Barça N, Özoran K (2009). T he relationship between enthesitis indices and disease activity parameters in patients with ankylosing spondylitis. Clin Rheumatol.

[24] Bijur PE, Silver W, Gallagher EJ (2001). Reliability of the visual analog scale for measurement of acute pain. Acad Emerg Med.

[25] Calin A, Garrett S, Whitelock H, Kennedy LG, O’hea J, Mallorie P (1994). A new approach to defining functional ability in ankylosing spondylitis: the development of the Bath Ankylosing Spondylitis Functional Index. J Rheumatol.

[26] Rostom S, Benbouaaza K, Amine B, Bahiri R, Ibn Yacoub Y, Ali Ou Alla S, Abouqal R, Hajjaj-Hassouni N (2010). Psychometric evaluation of the Moroccan version of the Bath Ankylosing Spondylitis Functional Index (BASFI) and Bath Ankylosing Spondylitis Disease Activity Index (BASDAI) for use in patients with ankylosing spondylitis. Clin Rheumatol.

[27] Vlietstra L, Stebbings S, Meredith-Jones K, Abbott JH, Treharne GJ, Waters DL (2019). Sarcopenia in osteoarthritis and rheumatoid arthritis: the association with self-reported fatigue, physical function and obesity. PLoS ONE.

[28] Taylor S, Frost H, Taylor A, Barker K (2001). Reliability and responsiveness of the shuttle walking test in patients with chronic low back pain. Physiother Res Int.

[29] Doward L, Spoorenberg A, Cook S, Whalley D, Helliwell P, Kay L (2003). Development of the ASQoL: a quality of life instrument specific to ankylosing spondylitis. Ann Rheum Dis.

[30] He Q, Luo J, Chen J, Yang J, Yao C, Xu C, Tao Q (2022). The validity and reliability of quality of life questionnaires in patients with ankylosing spondylitis and non-radiographic axial spondyloarthritis: a systematic review and meta-analysis. Health Qual Life Outcomes.

[31] Guermazi M, Ghroubi S, Kassis M, Jaziri O, Keskes H, Kessomtini W, Hammouda B, Elleuch MH (2006). Validity and reliability of Spinal Mouse to assess lumbar flexion. Ann Readapt Med Phys.

[32] Iguchi S, Inoue-Hirakawa T, Nojima I, Noguchi T, Sugiura H (2021). Relationships between stress urinary incontinence and trunk muscle mass or spinal alignment in older women. Low Urin Tract Symptoms.

[33] Wright A, Hannon J, Hegedus EJ, Kavchak AE (2012). Clinimetrics corner: a closer look at the minimal clinically important difference (MCID). J Man Manip Ther.

[34] Liang Z, Fu C, Zhang Q, Xiong F, Peng L, Chen L (2021). Effects of water therapy on disease activity, functional capacity, spinal mobility and severity of pain in patients with ankylosing spondylitis: a systematic review and meta-analysis. Disabil Rehabil.

[35] Bestaş E, Dündar Ü, Köken T, Koca B, Yeşil H (2022). The comparison of effects of balneotherapy, water-based and land-based exercises on disease activity, symptoms, sleep quality, quality of life and serum sclerostin level in patients with ankylosing spondylitis: a prospective, randomized study. Arch Rheumatol.

[36] Abadi FH, Sankaravel M, Zainuddin FF, Elumalai G, Choo LA, Sattari H (2020). A perspective on water properties and aquatic exercise for older adults. Int J Aging Health Mov.

[37] Dundar U, Solak O, Toktas H, Demirdal U, Subasi V, Kavuncu V (2014). Effect of aquatic exercise on ankylosing spondylitis: a randomized controlled trial. Rheumatol int.

[38] Zhao Q, Dong C, Liu Z, Li M, Wang J, Yin Y (2020). The effectiveness of aquatic physical therapy intervention on disease activity and function of ankylosing spondylitis patients: a meta-analysis. Psychol Health Med.

[39] Andonian BJ, Masi AT, Aldag JC, Barry AJ, Coates BA, Emrich K (2015). Greater resting lumbar extensor myofascial stiffness in younger ankylosing spondylitis patients than age-comparable healthy volunteers quantified by myotonometry. Arch Phys Med Rehabil.

[40] Konrad A, Reiner M, Thaller S, Tilp M (2019). The time course of muscle-tendon properties and function responses of a five-minute static stretching exercise. Eur J Sport Sci.

